# Mesenchymal stem cells in radiation-induced lung injury: From mechanisms to therapeutic potential

**DOI:** 10.3389/fcell.2022.1100305

**Published:** 2022-12-12

**Authors:** Guowen Hou, Jinjie Li, Wenyun Liu, Jinlong Wei, Ying Xin, Xin Jiang

**Affiliations:** ^1^ Jilin Provincial Key Laboratory of Radiation Oncology and Therapy, The First Hospital of Jilin University, and Key Laboratory of Pathobiology, Ministry of Education, Jilin University, Changchun, China; ^2^ Key Laboratory of Pathobiology, Ministry of Education, Jilin University, Changchun, China; ^3^ Department of Radiation Oncology, The First Hospital of Jilin University, Changchun, China; ^4^ NHC Key Laboratory of Radiobiology, School of Public Health, Jilin University, Changchun, China

**Keywords:** radiation-induced lung injury (RILI), mesenchymal stem cells (MSCs), gene modification, inflammatory response, cytokines, oxidative stress

## Abstract

Radiotherapy (RT) is an effective treatment option for multiple thoracic malignant tumors, including lung cancers, thymic cancers, and tracheal cancers. Radiation-induced lung injury (RILI) is a serious complication of radiotherapy. Radiation causes damage to the pulmonary cells and tissues. Multiple factors contribute to the progression of Radiation-induced lung injury, including genetic alterations, oxidative stress, and inflammatory responses. Especially, radiation sources contribute to oxidative stress occurrence by direct excitation and ionization of water molecules, which leads to the decomposition of water molecules and the generation of reactive oxygen species (ROS), reactive nitrogen species (RNS). Subsequently, reactive oxygen species and reactive nitrogen species overproduction can induce oxidative DNA damage. Immune cells and multiple signaling molecules play a major role in the entire process. Mesenchymal stem cells (MSCs) are pluripotent stem cells with multiple differentiation potentials, which are under investigation to treat radiation-induced lung injury. Mesenchymal stem cells can protect normal pulmonary cells from injury by targeting multiple signaling molecules to regulate immune cells and to control balance between antioxidants and prooxidants, thereby inhibiting inflammation and fibrosis. Genetically modified mesenchymal stem cells can improve the natural function of mesenchymal stem cells, including cellular survival, tissue regeneration, and homing. These reprogrammed mesenchymal stem cells can produce the desired products, including cytokines, receptors, and enzymes, which can contribute to further advances in the therapeutic application of mesenchymal stem cells. Here, we review the molecular mechanisms of radiation-induced lung injury and discuss the potential of Mesenchymal stem cells for the prevention and treatment of radiation-induced lung injury. Clarification of these key issues will make mesenchymal stem cells a more fantastic novel therapeutic strategy for radiation-induced lung injury in clinics, and the readers can have a comprehensive understanding in this fields.

## 1 Introduction

Radiotherapy (RT) is an effective treatment option for thoracic malignancies. Radiation-induced lung injury (RILI) is a serious complication of RT. RILI consists of two stages: the acute phase, usually manifesting as radiation pneumonitis (RP), and the chronic phase, manifesting as radiation pulmonary fibrosis (RPF). The symptoms of RILI are mainly dry cough, severe dyspnea, and respiratory failure, resulting in death (Rajan Radha and Chandrasekharan, 2017; Hanania et al., 2019). According to reports, 5%–20% of patients will experience dyspnea after RT, and pulmonary function tests often shows significant subclinical reduction. RT for cancer of the lung, mediastinal lymphatics, and breast exhibits different incident rate of RILI, respectively 5%–50%, 5%–10%, and 1%–5% (Marks et al., 2010; Zhang et al., 2010). Higher radiation dose, older age and larger tumor diameter are significant adverse risk of RILI. The therapeutic dose of radiation that can be given in effectively control cancer are restricted in patients due to RILI. So, the patients with RILI have poor prognosis (Kong et al., 2021). RILI is concerning because of its high incidence and potentially severe consequences. Multiple factors contribute to the progression of RILI, including genetic alterations, oxidative stress, and inflammatory responses. Immune cells and multiple signaling molecules play a major role in the entire process of RILI. The current therapies for RILI lack treatment plans and guidelines.

Mesenchymal stem cells (MSCs), as cell-based treatments, have the potential to treat RILI. MSCs are pluripotent stem cells that have multiple differentiation potentials and are derived from various tissues (Samsonraj et al., 2017). MSCs have been successfully isolated from many tissues, particularly marrow-derived MSCs, which are frequently used and have established criteria for characterization (Pittenger et al., 1999). MSCs can interact with cells of the innate and acquired immune systems and migrate to injured tissues, where they can inhibit the release of pro-inflammatory cytokines and promote the survival of damaged cells by regulating signaling molecules (Uccelli et al., 2008). Owing to their ability to regulate signaling, researchers are interested in using MSCs to treat RILI.

## 2 Changes in lung tissue in RILI

The lung parenchyma is composed of the respiratory bronchioles, alveolar ducts, and alveoli. The alveolar stroma is composed of fibroblasts, alveolar macrophages, and the extracellular matrix (ECM). When exposed to radiation, these tissues and cells of the lung suffer damage in genes, which is considered a direct effect of radiation. Ionizing radiation can disrupt atomic structures, damage normal DNA or RNA sequences, and affect biological functions (Liu et al., 2021). Another significant change is the radiation-induced water radiolysis. The water in cells and tissues can absorb radiation and energy. The process results in excitation and ionization of water, which leads to the generation of reactive oxygen species (ROS) and reactive nitrogen species (RNS) (Azzam et al., 2012). ROS and RNS are major damage factors that can influence the normal conditions of cells and tissues. Moreover, the expression of genes related to oxidation and anti-oxidation can be affected.

Under the influence of these initial damaging factors, multiple cellular events occur. Mitotic catastrophe and apoptosis lead to endothelial cell death mainly by activating p53 or by the sphingomyelin ceramide pathway. Subsequently, apoptosis causes the release of damage-associated molecular patterns and activation of the systemic inflammatory response (Wijerathne et al., 2021). Radiation can also cause cellular senescence. Induction to senescence is considered as an important mechanism in the treatment of cancer by RT, since it causes permanent growth arrest of DNA-damaged cells preventing their reproduction and stimulates the immune system to rapidly eliminate these genetically unstable cells. However, in addition to cancer cells, normal tissue cells in the lungs are also significantly affected by cell senescence, and the excessive secretion of senescence-associated secretion phenotype causes subsequent damage (He et al., 2019). Some recent studies have found that radiation can induced ferroptosis by promoting lipid peroxidation (Lei et al., 2020a).

**FIGURE 1:**
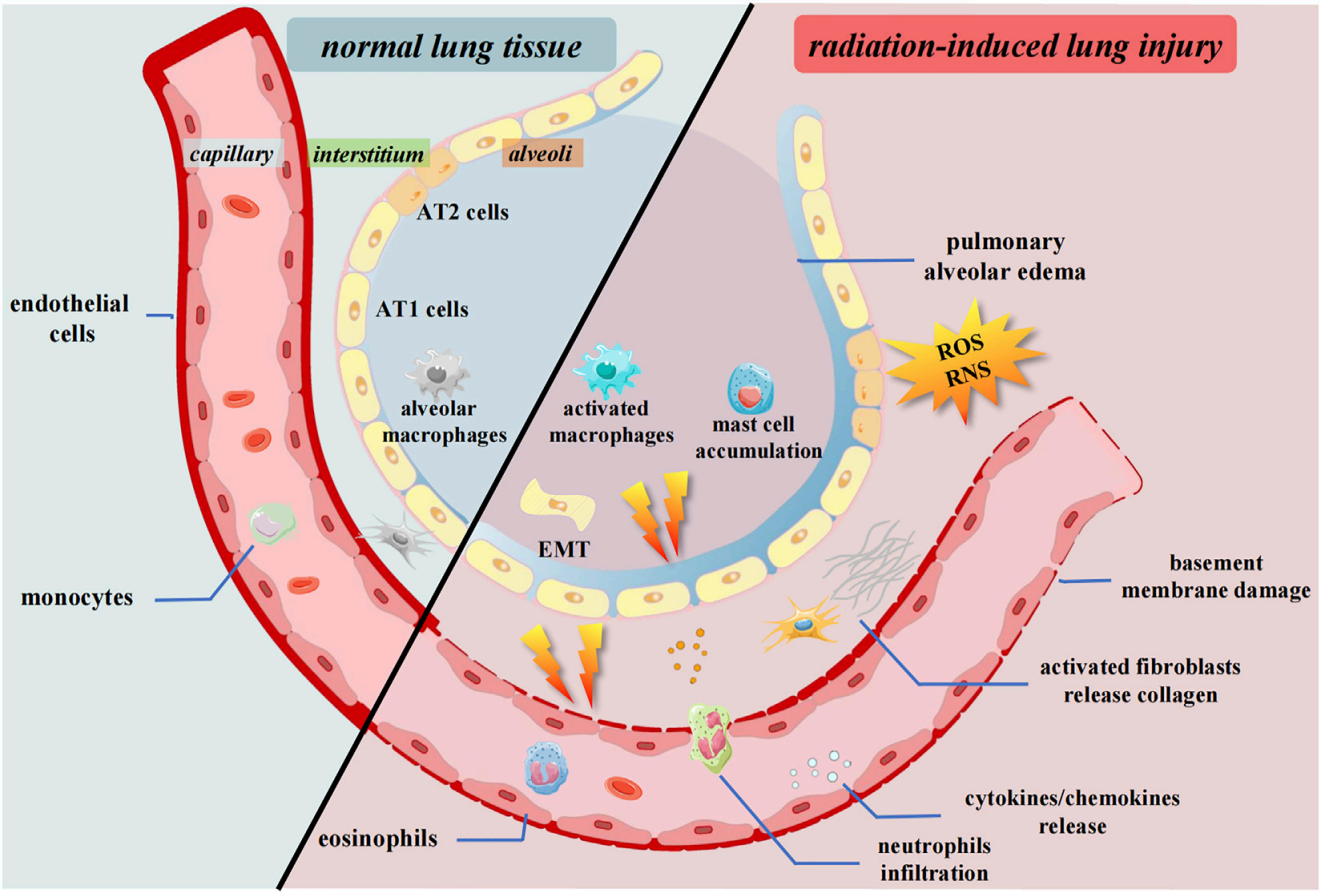
Pulmonary tissue changes after irradiation. Radiation causes the release of cytokines and chemokines. The inflammatory cells and fibrosis associated cells are activated and infiltrate to interstitium. ROS and RNS accumulate in the microenvironment. The epithelium undergoes EMT process. AT1 cells, Alveolar type 1 cells; AT2 cells, Alveolar type 2 cells; ROS, Reactive oxygen species; RNS, Reactive nitrogen species; EMT, Epithelial-mesenchymal transition.

**FIGURE 2:**
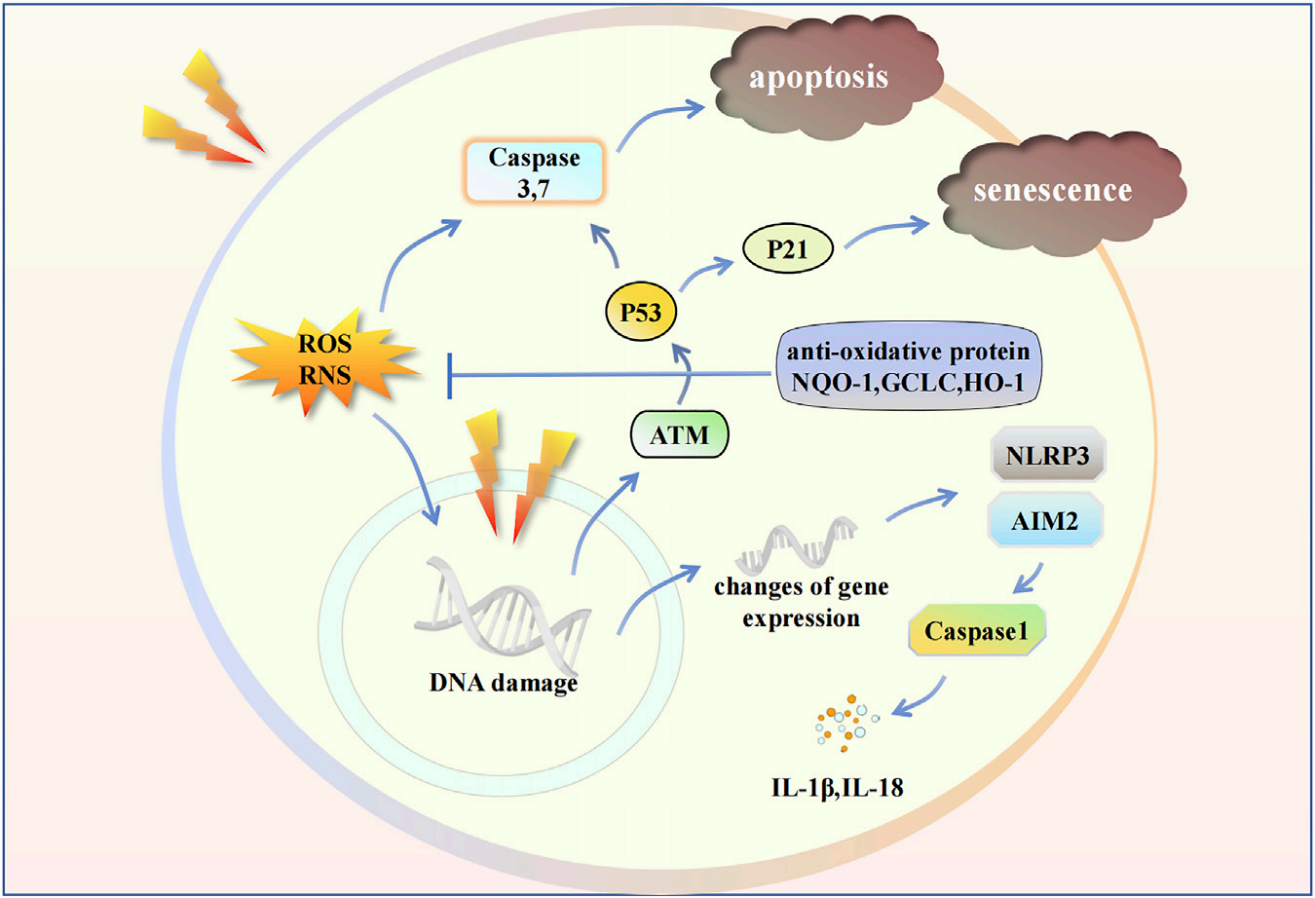
Radiation influences some intracellular signaling pathways in endothelial cell. Radiation leads to the changes in gene expression and damage in gene structure. ROS and RNS cause apoptosis. Inflammasomes induce the release of cytokines. The activation of ATM/P53/P21 signaling pathway cause cell senescence. IL, Interleukin; ATM, Ataxia telangiectasia mutated; P21, Cyclin-dependent kinase inhibitor 1A; NQO-1, NAD(P)H Quinone Dehydrogenase 1; GCLC, Glutamate-Cysteine Ligase Catalytic Subunit; HO-1, Heme oxygenase-1.

These changes lead to a subsequent inflammatory response. Inflammatory cells release large amounts of signaling molecules, leading to severe inflammation and immune system disorders (Giuranno et al., 2019; Wei et al., 2021). Subsequently, lung tissue fibrosis progresses with an increase in cytokine levels and the expression of collagen genes. These continuous stress factors go beyond the usual regulatory range of cellular and organismal homeostasis, eventually leading to pulmonary fibrosis (Petkau, 1987). This review elaborates on the progress of cells and organs and the adjustment of signaling molecules and immune systems in RILI (Figures 1, 2).

### 2.1 Changes in genes and expression

Radiation leads to direct DNA damage, including base deletions, and single- and double-strand breaks (Yan et al., 2022). The first protein activated by the DNA damage response is “ataxia telangiectasia mutated,” which can activate downstream P53 and P21, inducing cell senescence (Lei et al., 2020b). Moreover, DNA damage contributes to the inflammatory microenvironment and stimulates signaling molecules such as type 1 interferon (IFN) and interleukin (IL)-1 (Wang et al., 2020; Cinat et al., 2021). Radiation also inhibits transcriptional regulation, including mRNA processing, DNA-dependent regulation of transcription, chromatin modification, and RNA splicing (Mathew et al., 2011). Changes in the quantity and quality of gene expression may be the earliest events in the body’s response to radiation and this variation leads to subsequent biological processes (Thakur et al., 2021). Such genes are associated with organismal injury, cellular movement, immune cell trafficking, and inflammatory response. The upregulated genes were mainly *SERPINA3, ATP12A, LRRN4, GJB2*, and *CLDN10*, while the downregulated genes were *PNMT, TOX3, FAM107A, RXRG,* and *LPA*. The major upstream regulators of these genes are associated with angiotensinogen, IL-1, and transforming growth factor-β (TGF-β), indicating that these molecules play key roles in the progression of RILI. Radiation also causes discrepancies in circRNAs and miRNAs in contrast to normal pulmonary tissues (Li et al., 2021). The main target mRNAs of these differentially expressed circRNAs and miRNAs are related to T cell proliferation, chemokine receptor activity, and the B-cell receptor signaling pathway. Thus, the migration of inflammatory cells and the release of cytokines, along with the immune response, play a critical role after radiation. Although most current studies are based on mouse models, there are some studies on RILI in non-human primate (NHP) models, which are much similar to human than rodents. The microarray expression profiling of NHP models shows that the differentially expressed genes are mainly related to expression of inflammatory cytokines, complement factors, apoptosis-related molecules (Cui et al., 2021).

### 2.2 Changes in oxidation and anti-oxidation

The continuous production of ROS after RT affects the homeostasis of progeny cells and bystander cells, resulting in a wide range of oxidative damage, including protein carbonylation and lipid peroxidation, which increases the chance of spontaneous gene mutations and neoplastic transformation (Buonanno et al., 2011a; Buonanno et al., 2011b; Shen et al., 2018). These biological processes primarily damage genes and cause cellular dysfunctions. ROS can lead to the generation of oxidative lipids in the pulmonary endothelial cells. Oxygenated lipids in endothelial cells, as major biomarkers, can reflect the damage process of damage. It also correlates with signaling pathways involved in cell proliferation, apoptosis, programmed cell death, inflammation, and immune surveillance (Tang et al., 2002; Cole et al., 2003; Greenberg et al., 2006; Haworth and Levy, 2007). Research has shown that cardiolipin and phosphatidylserine are the main phospholipids that are significantly oxidized (Tyurina et al., 2011). Caspase 3/7 activity is accompanied by the degree of oxidation of cardiolipin and phosphatidylserine after radiation. These oxidative phospholipids may trigger apoptosis. Apart from the influence of ROS, some major oxidative damage of RILI comes from RNS, which includes nitric oxide (NO) and peroxynitrite. NO is a free radical that can chain react with superoxide, producing the potent oxidizing agent peroxynitrite. NO and peroxynitrite can induce lipid peroxidation in biological membranes, nitration, and hydroxylation of aromatic acid residues, and sulfhydryl oxidation of proteins (Giaid et al., 2003). They mainly influence the alveolar epithelial cells and specific surfactant apoproteins. When radiation-induced oxidative stress damage occurs, the body must enhance its antioxidant biological function to defend itself against such damage. Nuclear factor E2-related factor 2 (Nrf2) is an effective cellular antioxidant protein that triggers a cascade of downstream antioxidant signals. Nrf2 is upregulated after irradiation and increases the expression of antioxidant response elements, which mediate phase II antioxidant enzymes in pulmonary tissue (Lee et al., 2007; Mathew et al., 2013). Genes related to antioxidant function are activated, including *NQO1*, *GCLC*, *HO–1*, *Gpx1*, *Prdx1*, and *Txn1*. Recently, researchers discovered that the gene *MIF* and its encoding molecule, macrophage migratory inhibition factor, a multifunctional pleiotropic cytokine, had a positive relationship with Nrf2 and downstream antioxidized enzymes (Mathew et al., 2013).

### 2.3 Changes in cytokines, inflammasomes, and immune cells

When the lung tissue is irradiated, an inflammatory response occurs. The alveolar epithelium and vascular endothelium are damaged. Simultaneously, due to increased microvascular permeability, immune cells are recruited to the injured site by chemokines. Many immune cells are activated to produce inflammatory signaling molecules, which lead to the initiation of additional paracrine and autocrine loops between several cells (Hanania et al., 2019).

Cytokines, as the most important biomarkers, are considered to predict the progression of RILI. Radiation can cause changes in multiple cytokines, including IL, IFN-γ, TGF-β, tumor necrosis factor-α (TNF-α), granulocyte colony-stimulating factor (G-CSF), and granulocyte macrophage colony-stimulating factor (GM-CSF). Most of these cytokines are associated with RILI (Ao et al., 2009). Inflammatory cytokines are key mediators of the pathogenesis of RP. The levels of pro-inflammatory cytokines in the serum, such as IL-1α and IL-6, are significantly elevated during all stages of RILI. Subsequently, the same changes in IL-6 were confirmed in bronchoalveolar lavage fluid (Chen et al., 2001). IL-7, IL-12, IL-15, IL-16 were also found to be significantly changed at different time points after radiation in NHP model (Cui et al., 2020). These changes in cytokines can persist for several months after completing RT, which illustrates that these signaling molecule events extend the damage response (Barthelemy-Brichant et al., 2004). Radiation can elevate TNF-α expression in lung tissue (Rübe et al., 2002). TNF-α, as a proinflammatory cytokine, can stimulate the expression of adhesion molecules to recruit leukocytes and induce the production of prostaglandins and other mediators of inflammation. Besides inflammatory cytokines, other cytokines also vary prominently during RILI. Radiation can upregulate TGF-β expression, leading to RILI progression (Rube et al., 2000). TGF-β plays a significant role in regulating inflammatory cell infiltration, collagen deposition, autophagy, apoptosis, and EMT, and is a key molecule in the process of fibrogenesis in cells and ECM (Ikushima and Miyazono, 2010; Eser and Jänne, 2018). TGF-β, an upstream regulator, can regulate many molecules in the TGF-β/Smad pathway, such as α-smooth muscle actin, collagen I, and tissue inhibitors of matrix metalloproteinases (Meng et al., 2016). Multiple regulators targeting TGF-β can alleviate RILI (Gauter-Fleckenstein et al., 2010; Xue et al., 2013; Kim et al., 2020; Ying et al., 2021). Lipoxin A4, a bioactive product of arachidonic acid, can target the TGF-β/Smad pathway to exert anti-inflammatory and antifibrotic properties in RILI (Kim et al., 2020). Pirfenidone and MnTE-2-PyP5+ also alleviated RILI by regulating TGF-β (Gauter-Fleckenstein et al., 2010; Ying et al., 2021). G-CSF and GM-CSF levels in the lungs are elevated after radiation (Ao et al., 2009). G-CSF is responsible for activating and recruiting neutrophils to the lung, which may contribute to pulmonary damage (Gandhi et al., 2019). GM-CSF functions similar to G-CSF and stimulates the activation and proliferation of macrophages, which prompts the progression of inflammation (Shiomi and Usui, 2015). However, some researchers believe that GM-CSF exerts a protective effect against RILI. GM-CSF can maintain normal pulmonic homeostasis and it is essential for biological immunity processes (Hu et al., 2020). Hence, its role in RILI is not yet well understood. Chemokines also influence RILI. Chemokine C-C motif ligand (CCL) is associated with epithelial senescence and vascular dysfunction after radiation (Wiesemann et al., 2019). Radiation increases CCL2 levels, which contributes to the immune regulation and migration of myeloid cells. In addition, thoracic irradiation caused CCL3 and CCL8 production *in vivo*. These chemokines bind to CC chemokine receptors (CCR), which promote injury, inflammation, and mortality by driving leukocyte recruitment (Yang et al., 2011; Kim et al., 2020).

Inflammasomes have recently been found to play a crucial role in RILI. Inflammasomes can influence the release of certain cytokines. One such inflammasome, AIM2, can sense potentially dangerous cytoplasmic DNA with its oligonucleotide/oligosaccharide-binding domain, and it can interact with apoptosis-associated speck-like protein through its amino-terminal pyrin domain to activate caspase-1 (Fernandes-Alnemri et al., 2009). A study found that radiation caused AIM2 translocation to the nucleus to sense damaged double-stranded DNA, followed by AIM2 oligomerization, assembly, and activation (Gao et al., 2019). Subsequently, caspase-1 is then activated, and IL-1β is released abundantly, triggering inflammation. Some researchers have revealed that the NLRP3 inflammasome is increased by irradiation. Subsequently, mature cleaved capase-1 was significantly upregulated and the proportion of IL-1β and IL-18 was dramatically elevated, which led to a more severe inflammatory response in the later stage (Wu et al., 2019).

Radiation leads to increases not only in the total immune cell count, but also in the category and quantity of immune cells undergo a series of changes. Immune cells, including polymorphonuclear leukocytes, lymphocytes, macrophages, and mast cells, accumulate in alveoli after radiation (Majori et al., 2000; Cappuccini et al., 2011; Kunwar et al., 2013). Interstitial macrophages decreased, while alveolar macrophages significantly increased, indicating macrophage migration. Macrophages can transform into activated macrophages with the pro-inflammatory M1 phenotype. Massive infiltration of activated neutrophils in the lungs induces thickened alveolar septa (Abernathy et al., 2015), and the total number of activated T lymphocytes increases continuously. Radiation also affects the composition of the T lymphocyte subsets recruited to the lungs. The proportions of T helper (Th) 17 cells and regulatory T cells (Tregs) increase greatly day 21 post irradiation (Cappuccini et al., 2011). Tregs are thought to possess different functions in the RP and RPF stages. Tregs are recruited by CCL2 and inhibit inflammatory responses in RP. However, Tregs contribute to RPF through several mechanisms, including promotion of fibrocyte accumulation and EMT, modulation of the Th1/Th2 balance, and suppression of Th17 responses (Guo et al., 2020).

### 2.4 Changes in tissue composition and organ function

Changes in elements of tissues often occur after radiation exposure. Alveolar type 1 (AT1) cells on the alveolar surface undergo apoptosis after radiation, while alveolar type 2 (AT2) cells accelerate proliferation to fill the defect. Moreover, the proliferation of AT2 cells and an unbalanced AT1:AT2 ratio leads to more pulmonary surfactant being produced. Exposing epithelial cells to radiation results in the rapid induction of a stress response in which pro-inflammatory cytokines are produced and immunological reactions take place in the airways (Rübe et al., 2005). Radiation activates the expression of fibrosis-related genes, resulting in changes in ECM composition, especially increased collagen (Lei et al., 2020b). In normal situations, the ECM of the alveolar wall is mainly composed of proteoglycans, fibronectin, laminin, entactin, and types IV and VII collagens. When exposed to radiation, these proportions will change in quality and quantity. For example, type I/III/IV collagens increase significantly, especially type IV collagen (Tsoutsou and Koukourakis, 2006). Growing evidence suggests that radiation can prompt alveolar epithelial cells to undergo epithelial-mesenchymal transition (EMT) (Kim et al., 2006). EMT is considered crucial to the pathogenesis of organ fibrosis (Hewlett et al., 2018; Wang et al., 2020). Epithelial cells acquire mesenchymal-like properties essential for the progressive accumulation of collagen and extracellular matrix proteins, ultimately leading to lung fibrosis. Furthermore, endothelial cells are affected by pulmonary dysfunction. The pulmonary endothelial cells as protective barriers of blood-vessel walls are prominently lost, and the basement membrane of endothelial cells ruptures at an early stage (Ghobadi et al., 2012). Owing to the enlarged endothelial gap, inflammatory infiltrate diffusely expands resulting in abnormal lung radiation density on CT scans (Thakur et al., 2021). Endothelial cells serve as a regulator of normal physiological function for their inducible metabolic and synthetic function. With endothelial cell loss, the medial layer becomes muscularized, the adventitia becomes thicker, and damage to the neointima occurs. This indicates the pulmonary vasculature is remodeled during RILI. Irradiation often induces radiation dose-dependent pulmonary hypertension due to changes in the pulmonary vessels. Respiration function is impaired by radiation; in particular, the pulmonary diffusion capacity decreases significantly (Elliott et al., 2019). These changes in normal pulmonary physiology are associated with the clinical symptoms of RILI.

## 3 MSCs alleviate RILI

Current treatments are not effective for RILI, and have many potential side effects. The treatments of RPF are primarily supportive, such as supplemental oxygen to relieve the symptoms (Hanania et al., 2019). The current clinical treatment of RP mainly involves high doses of corticosteroids and other anti-inflammatory drugs (Bledsoe et al., 2017). However, these treatments have severe problems, including inefficiency, recurrence of symptoms, and complications due to use of steroids (Sekine et al., 2006; Grennan and Wang, 2019). Several combined treatments for RILI exist, but none has been satisfactory (Raghu et al., 2012). Given the unique characteristics of MSCs, some researchers have utilized them as a potentially revolutionary treatment for RILI.

**FIGURE 3:**
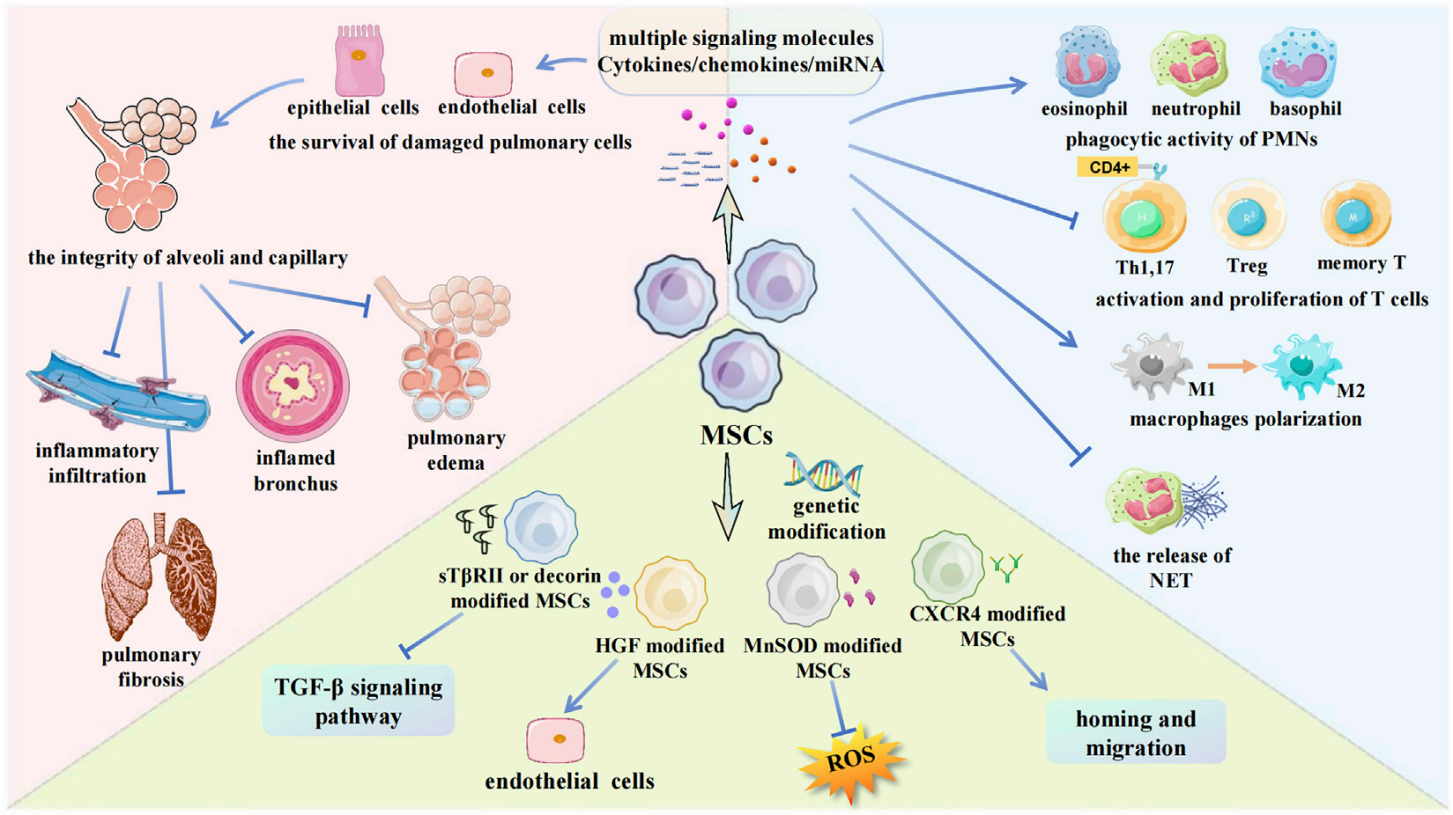
Some mechanisms about MSCs for RILI in current researches. The main methods to apply MSCs in RILI are showed in the picture. MSCs can regulate multiple signaling molecules, which protect pulmonary cells. MSCs inhibit leukocytes infiltration and regulate leukocytes function. Genetically modified MSCs can strengthen target gene expression. PMNs, Polymorphonuclear leukocytes; Treg, Regulatory T cell; sTβRII, TGF-β type II receptor; HGF, Hepatocyte growth factor; MnSOD, Manganese superoxide dismutase; CXCR4, CXC-chemokine receptor 4; TGF-β, Transforming growth factor beta.

MSCs are easy to obtain, genetically stable, have low immunogenicity, and can easily pass through the immune barrier (Lee et al., 2011). The differentiation and grafting of MSCs are thought to play a substantial role in repairing damaged tissues (Prockop, 1997; Sueblinvong et al., 2008). MSCs can migrate along vascular networks. Because of their near-ubiquitous perivascular locations and rich network of projections, MSCs are ideal first responders to inflammation (Soliman et al., 2021). For RILI, inflammatory changes in the immune system occur both in the early stage and during the entire process. Therefore, MSCs as initial damage sensors and inhibitors of innate inflammation can react to changes in metabolism and microenvironment, including cytokine secretion, signaling molecule release, and related immune cell regulation (Harrell et al., 2019). The specific mechanisms associated with MSC therapy for RILI are shown (Figure 3). There are two generations of MSCs treatment for RILI. The first generation, direct administration of MSCs is a basic and effective method (Table 1), and the second generation involves modifying MSCs to act as gene therapy vehicles. Extracellular vesicles (EVs) secreted by MSCs and the culture medium of MSCs are found to have similar functions in preventing pulmonary damage. Therefore, researchers have occasionally used them to treat RILI (Xue et al., 2013; Lei et al., 2020b). The function of EVs is depend on their cargo, which can include functional mRNA, small RNA, lipids, and proteins. For example, EVs as miRNA delivery vehicles can inhibit wound inflammation by delivering miRNA21, miRNA146a, miRNA181 (Ti et al., 2016). The culture medium of MSCs is a mixture of several hundred to thousands of different proteins, cytokines, growth factors, and enzymes, which is secreted by MSCs. EVs are also included in the mixture. It is difficult to clarify all factors that play roles in mitigating the damage. Some researchers aimed to compare the treatment effects of different method. In the study of high fat-induced cardiac complications, MSCs can significantly regulate the TGF-β signaling pathway. However, culture medium has a certain activation effect on transcriptional factors suppressor of cytokine signaling 3 and peroxisome proliferator activated receptor gamma, indicating that MSCs and culture medium may have similar therapeutic effects, but their mechanisms may be different (Daltro et al., 2017). Other studies about MSCs and EVs show the same result (Huang et al., 2016). It cannot be denied that EVs and culture medium as a cell-free therapy offers more advantages, such as suitable for long-term storage, no heterologous risk. There are also some flaws in EVs and culture medium, such as no standard of isolation method (Cheng et al., 2020). In general, both EVs and culture medium are derived from MSCs. Therefore, it is difficult to decide which method is better. Compared to current drug treatment, MSCs have good prospects as a new treatment strategy.

**TABLE 1:** Current and ongoing studies on direct administration of MSCs in RILI.

Researches	Model	The source of MSCs	Administration	The signaling pathways or molecules affected by MSCs	Research conclusions
Li et al. (2022)	C57BL/6 mice	Mouse bone marrow	iv	MSCs inhibit AKT/GSK3β pathway by miRNA-466f-3p	MSCs possess anti-fibrotic properties and prevent radiation-induced EMT
Lei et al. (2020b)	C57BL/6 mice	Human placenta	iv	MSCs downregulate ATM/P53/P21 pathway by miR-214-3p	EVs derived from MSCs can attenuate the radiation-induced injury of endothelial cells
Shao et al. (2021)	Male BALB/c mice	Human adipose tissue	iv	MSCs enhance DKK-1 to inhibit Wnt/β-catenin pathway	MSCs inhibit pulmonary fibrosis and EMT
Zhang et al. (2019a)	Sprague-Dawley rats	Rat adipose tissue	iv	MSCs inhibit TGF-β and α-SMA	MSCs can effective reduce lung fibrosis and inflammation when twice delivered at the 2 h and 7 days
Hao et al. (2018)	Male beagle	Human umbilical cord	Intratracheal transplantation	MSCs inhibit TGF-β/smad pathway	MSCs can reduce oxidative stress, inflammatory reactions thereby reducing lung injury
Klein et al. (2017)	C57BL/6 mice	Mouse aortas and mouse bone marrow	iv	MSCs restore SOD1 level	MSCs can counteracts radiation-induced vascular damage
Perez et al. (2017)	Female Sprague-Dawley rats	Rat bone marrow	iv and endotracheal injection	No pathways or molecules are studied. Research aims at understanding of the biological location and the mechanisms of action of MSCs after being injected	MSCs injected *via* endotracheal administration can hold higher number in lung
Xia et al. (2016)	Mice	Human bone marrow	iv	MSCs reduce the level of IL-10 and increase the level of TNF-α	Low-dose MSCs contribute to functional recovery after radiation
Klein et al. (2016)	C57BL/6 mice and BALB/c mice	Mouse aortas and mouse bone marrow	iv	MSCs inhibit Mmp2	MSCs protect the lung tissue from radiation-induced vascular dysfunction
Zhang et al. (2015)	Human lung fibroblasts	Human umbilical cord	Transwell co-culture of fibroblasts with MSCs	MSCs attenuate Wnt/β-catenin pathway	MSCs attenuate the radiation-induced activation in fibroblasts
Jiang et al. (2015)	Sprague-Dawley rats	Rat adipose tissue	iv	MSCs reduce serum level of IL-1, IL-6, TNF-α and increase the level of IL-10	MSCs attenuate acute RILI by anti-inflammation, anti-fibrosis and anti-apoptosis mechanisms
Wang et al. (2014)	Male Wistar rats	Human umbilical cord	iv	No pathways or molecules are studied only histopathological observation	MSCs have definite therapeutic effects on RP in rats

IV, intravenous; MSC, mesenchymal stem cell; GSK3β, glycogen synthase kinase 3β; EMT, epithelial-mesenchymal transition; ATM, ataxia telangiectasia mutated; EVs, extracellular vesicles; P21, cyclin-dependent kinase inhibitor 1A; DKK-1, Dickkop-1; TGF-β, transforming growth factor β; α-SMA, α-smooth muscle actin; SOD1, superoxide dismutase 1; IL, interleukin; TNF-α, tumor necrosis factor α; Mmp2, matrix metalloproteinase 2; RILI, radiation-induced lung injury; RP, radiation pneumonitis.

### 3.1 Targeting signaling molecules with MSCs

MSCs can directly secrete the anti-inflammatory cytokines IL-10 and IL-2 in an inflammatory environment (Jiang and Xu, 2020). These anti-inflammatory factors directly guide the immune system to exert anti-inflammatory effects and reduce pro-inflammatory cytokines (Zhang et al., 2019a). MSCs also regulate the expression of most inflammatory cytokines. IL-1, the main mediator of innate immunity and inflammation, is significantly reduced after MSC treatment (Garlanda et al., 2013). Moreover, the gene expression of pro-inflammatory cytokines such as *IL-6* and *TNF-α* is significantly decreased. As a multifunctional cytokine, TGF-β can be regulated by MSCs in the RILI model, which means that MSCs also influence collagen deposition and EMT to ameliorate fibrosis (Jiang et al., 2015). Researchers have found that MSCs inhibit the canonical Wnt/β-catenin pathway by secreting Dickkopf-1. Dickkopf-1 can prevent the activation of low-density lipoprotein receptor-related protein 6, a type of Wnt3a receptor on the cell membrane, blocking the downstream signal transduction of Wnt (Shao et al., 2021). MSCs can regulate the migration of immune cells by influencing chemokines or receptors. MSCs can normalize the expression of CCL2, decreasing the directional migration ability of inflammatory cells (Klein et al., 2016). Moreover, the abnormally high proportion of monocytes and granulocytes observed during RILI was reversed by MSCs. Some evidence has shown that MSCs can alleviate RILI through miRNAs. miRNA-214-3p was discovered in EVs secreted by MSCs (Lei et al., 2020b). miRNA-214-3p suppresses endothelial cell damage and enhances radioprotective functions. MSC-derived miRNA-466f-3p could reverse EMT to inhibit fibrosis (Li et al., 2022). The possibility of species variation in the application of MSCs is considered. MSCs can reduce TNF-α and IL-1 in lung tissues and increase the partial pressure of blood oxygen in canines (Hao et al., 2018). Pathological examination also demonstrated that MSCs inhibited inflammatory cell infiltration, accompanied by collagen formation.

### 3.2 MSCs relieving RILI by regulating immune cells

Several studies have highlighted the critical role of MSCs in the infiltration of polymorphonuclear leukocytes. MSCs can potently suppress the activation and infiltration of neutrophils in the lung (Hong et al., 2017; Fang et al., 2018; Su et al., 2018). MSCs can direct polymorphonuclear leukocytes to ameliorate inflammation *via* enhanced phagocytic activity (van Dalen et al., 2019). The phagocytosis of polymorphonuclear leukocytes can clear debris and cause self-apoptosis. Then, these leukocytes are ingested by macrophages, which decrease the inflammatory response. Moreover, MSCs can drive neutrophils away from the site of inflammation and injury, which is also called the reversal of inflammatory infiltration (Shang et al., 2020). Neutrophil extracellular traps are network structures composed of chromatin, mitochondrial DNA, and proteins, which are secreted by neutrophils to resist the invasion of harmful substances. However, the overproduction of these traps causes severe tissue pathology in the context of inflammation (Rosazza et al., 2020). Researchers have found that MSCs effectively inhibit the ability of neutrophils to release these traps, which helps relieve inflammation (Navas et al., 2018).

Lymphocytes promote RILI progression, especially during the early inflammatory stage. MSCs exhibit powerful lymphocyte modulatory properties. Ren et al. (2008) found that MSCs can secrete chemokines including CCL5 and CXC-chemokine ligand 9. These chemokines can bind to CCR5 and CXC-chemokine receptor (CXCR) 3 on the surface of T cells, attracting T cells to migrate to the vicinity of MSC. MSCs express nitric oxide synthase or indoleamine 2,3-dioxygenase, which inhibit the activation and proliferation of adjacent T cells, leading to low adaptive immunity. Researchers have shown that MSCs can simultaneously inhibit the proliferation of central memory T cells and effector memory T cells, demonstrating their powerful ability to reduce inflammatory symptoms (Luque-Campos et al., 2019). MSCs can suppress the proliferation, activation, and differentiation of Th1 and Th17 cells when added at the early stages of differentiation (Luz-Crawford et al., 2013). Th1 and Th17 cell subsets are well known to promote inflammation (Lafaille, 1998; Bettelli et al., 2007). MSCs can also promote Treg differentiation through direct contact or a paracrine effect (Selmani et al., 2008; Ghannam et al., 2010). Treg cells can extensively inhibit the immune response and reduce inflammatory damage to the body.

Some researchers have focused on the EVs secreted by MSCs. Lei et al. (2020b) found that EVs secreted by MSCs can effectively reduce the retention of macrophages and neutrophils in lung tissues and the expression of inflammatory cell migration-related genes. Macrophage polarization is a key factor in RILI. MSCs can guide the polarization of macrophages *via* EVs (Lo Sicco et al., 2017). Then, the macrophages switch from the M1 to M2 phenotype, which means that M1 polarization-related secretion of IL-12 and TNF-α is reduced. Anti-inflammatory factors such as IL-10 are increased. In addition, MSCs cultured in a hypoxic environment seem to be more effective in regulating macrophage polarization.

### 3.3 Genetically modified MSCs for RILI

MSCs can trigger endogenous repair pathways, particularly the regulation of immune cells in RILI. Another ability of MSCs is that they can home to the damaged site when an injury occurs. Cytokines secreted from damaged site have been demonstrated to stimulate the migration of MSCs *in vitro* and induce their pathotropism *in vivo* which is mainly based on the activation of downstream migration-related signaling cascades. These signaling pathway mainly includes the phosphoinositide 3-kinase/AKT, stromal cell-derived factor-1 and its receptor chemokine CXCR4. Some miRNAs also determine the ability of migration (He and Zhang, 2019). Radiation can recruit MSCs to the damaged sites. Perez et al. (Perez et al., 2017) observed an apparent increase in the number of MSCs in the lungs after irradiation. Another study demonstrated that MSCs directly migrated to damaged pulmonary tissues after radiation by fluorescence endomicroscopy imaging. Moreover, irradiation prolongs the resident time of MSCs (Wang et al., 2013). Because MSCs possess the homing ability, an increasing number of researchers have used MSCs as a tool for gene therapy. They induce functional gene expression in MSCs. MSCs can then migrate to the injured site and express specific genes. Such MSCs are considered a superior to normal MSCs. Some studies on MSCs as gene vehicles for the treatment of RILI are summarized in Table 2.

**TABLE 2:** Current and ongoing studies on MSCs as gene vehicles for the treatment of RILI.

Researches	Model	The source of MSCs	Route	The signaling pathways or molecules affected by MSCs	Overexpressed molecules	Research conclusions
Zhang et al. (2019b)	Female C57BL/6 mice	Human umbilical cord	iv	CXCR4 genetically modified MSCs can decrease the level of SDF-1, TGF-β, α-SMA.	CXCR4	CXCR4-modified MSCs enhance the protection against RILI by homing and reparative effects
Liu et al. (2018)	Male C57BL/6 mice	Human umbilical cord	iv	Decorin-modified MSCs are more effective in inducing IFN-γ expression and inhibiting collagen	Decorin	Decorin-modified MSCs attenuate acute inflammation after irradiation and significantly inhibit later fibrosis
Chen et al. (2017)	Male mice	Human bone marrow	iv	MnSOD-modified MSCs influence the level of IL-1β, IL-6, TNF-α and TGF-β	MnSOD	MnSOD-modified MSCs significantly attenuate lung inflammation, ameliorate lung damage, and protect the lung cells from apoptosis
Wang et al. (2013)	Female mice	Human bone marrow	iv	HGF-modified MSCs influence the expression of IL-6, TNF-α, ICAM-1, TGF-β, Col1a1	HGF	HGF-modified MSCs improve histopathological and biochemical markers of lung injury
Xue et al. (2013)	Female C57BL/6 mice	Mouse compact bone	iv	Ex-TβRII genetically modified MSCs influence the level of α-SMA and CTGF	Ex-TβRII	Ex-TβRII genetically modified MSCs obviously alleviate lung injury, as reflected by survival and histopathology data

IV, intravenous; CXCR4, CXC-chemokine receptor 4; MSC, mesenchymal stem cell; RILI, radiation-induced lung injury; SDF-1, stromal cell-derived factor-1; TGF-β, transforming growth factor β; α-SMA, α-smooth muscle actin; IFN-γ, interferon-γ; MnSOD, manganese superoxide dismutase; IL, interleukin; TNF-α, tumor necrosis factor α; ICAM-1, intercellular adhesion molecule-1; Col1a1, collagen type I α1; α-SMA, α-smooth muscle actin; CTGF, connective tissue growth factor.

Genetically modified MSCs expressing TGF-β type II receptors have been under investigation to treat RILI (Xue et al., 2013). The receptor secreted by MSCs competes with the extracellular domain of TGF-β type II receptor, resulting in the inhibition of active TGF-β in plasma. Downstream signal transduction related to fibrosis was suppressed accordingly. MSCs protected pulmonary tissue, especially AT2 cells, *via* a paracrine mechanism. Moreover, genetically modified MSCs can reduce alveolar damage more efficiently than MSCs. Decorin is a natural inhibitor of TGF-β, and decorin-expressing MSCs can cure RILI (Liu et al., 2018). In particular, these modified MSCs also restrict the initiation and progression of fibrosis by downregulating Tregs.

In various injury and disease models, hepatocyte growth factor (HGF) improves cell survival, promotes tissue regeneration, and suppresses chronic inflammation and fibrosis (Nakamura et al., 2011). Wang et al. (2013) found that high HGF significantly reduced alveolar hemorrhage when *Hgf* genetically modified MSCs were under investigation to treat RILI. The expression of profibrotic genes, such as *TGF-β*, *Col1a1*, and *Col3a1* is reduced in the lung. High levels of HGF can also stimulate the release of sphingosine kinase, leading to the production of sphingosine-1-phosphate, a lipid messenger. When the messenger binds to its receptor, downstream signaling pathways associated with the regulation of immune cell trafficking and endothelial barrier function are activated (Matloubian et al., 2004; Sanna et al., 2006). Thus, genetically modified MSCs can ameliorate pulmonary damage in RILI.

Damage and senescence of endothelial cells and microvessels are closely related to RP and RPF. Restoring the expression level of superoxide dismutase (SOD) 1 can increase the viability of endothelial cells to alleviate RILI (Epperly et al., 1999; Holley et al., 2011; Klein et al., 2017). Chen et al. (2017) found that MSCs modified with the manganese *SOD* gene could attenuate capillary rupture, prevent telangiectasia, and maintain the integrity of microvessels. It can also regulate oxidative stress, which is highly associated with abnormal metabolism during RILI.

Currently, the stromal cell-derived factor-1/CXCR4 axis is an important signaling pathway related to the recruitment of MSCs to damaged sites (Wynn et al., 2004; Dar et al., 2005). Some researchers have constructed an MSC cell line that highly expresses CXCR4 (Zhang et al., 2019b). They found that CXCR4 overexpression enhanced the homing and migration abilities of MSCs *in vivo*. With increased MSC numbers in the lung tissue, morphological lung damage was obviously alleviated. This result again proves the efficacy of modified MSCs for the treatment of RILI.

## 4 Discussion

Multiple factors contribute to RILI progress, including changes in lung tissue, genetic alterations, oxidative stress, and inflammatory responses. MSCs exhibit powerful anti-inflammatory, anti-oxidative, and signaling effects. MSCs are considered a promising option for the treatment of RILI. In particular, therapies based on MSCs have been combined with gene modification. This new method can enhance the proliferative, migratory, and secretory properties of MSCs, providing further possibilities for RILI. However, applying MSCs still has some limitations, including difficulties in definite characterization of MSCs, and the varying properties of different sources of MSCs. Some studies also reported MSCs may not necessarily provide amelioration of fibrogenic disease in every case. Despite the considerable number of studies that have been performed, clinical management of RILI using the MSCs requires further investigation. For example, which secretory phenotype of MSCs plays a major role in their therapeutic process, and whether the MSCs responds to the RILI according to its stage of progression. Thus, the use of MSCs for the treatment of RILI requires additional data, both *in vitro* studies and in clinical investigations.

## Abbreviations

AT1, Alveolar type 1 cell; AT2, Alveolar type 2 cell; BAL, Bronchoalveolar lavage; ECM, Extracellular matrix; CCL, Chemokine C-C motif ligand; CCR, CC-chemokine receptor; CXCR, CXC-chemokine receptor; EMT, Epithelial-mesenchymal transition; EVs, Extracellular vesicle; G-CSF, Granulocyte colony-stimulating factor; GM-CSF, Granulocyte macrophage colony-stimulating factor; IFN, Interferon; IL, Interleukin; MSCs, Mesenchymal stem cell; NO, Nitric oxide; Nrf2, Nuclear factor E2-related factor 2; RILI, Radiation-induced lung injury; RNS, Reactive nitrogen species; ROS, Reactive oxygen species; RP, Radiation pneumonitis; RPF, Radiation pulmonary fibrosis; RT, Radiotherapy; TGF-β, Transforming growth factor-β; Th, T helper cell; TNF-α, Tumor necrosis factor-α; Treg, Regulatory T cell.
